# Salivary uric acid as a noninvasive biomarker of metabolic syndrome

**DOI:** 10.1186/1758-5996-4-14

**Published:** 2012-04-19

**Authors:** Maria Soukup, Izabela Biesiada, Aaron Henderson, Benmichael Idowu, Derek Rodeback, Lance Ridpath, Edward G Bridges, Andrea M Nazar, Kristie Grove Bridges

**Affiliations:** 1 Department of Biomedical Sciences, West Virginia School of Osteopathic Medicine, 400 N Lee St, Lewisburg, WV, USA; 2 Current affiliation, Department of Internal Medicine, San Antonio Uniformed Services Health Education Consortium, San Antonio, TX, USA; 3 Department of Assessment and Educational Development, West Virginia School of Osteopathic Medicine, Lewisburg, WV, USA; 4 Department of Clinical Sciences, West Virginia School of Osteopathic Medicine, Lewisburg, WV, USA

**Keywords:** Saliva, Biomarker, Metabolic syndrome, Uric acid

## Abstract

**Background:**

Elevated serum uric acid is associated with obesity, hypertension and metabolic syndrome. Because a linear relationship exists between serum and salivary uric acid (SUA) concentration, saliva testing may be a useful noninvasive approach for monitoring cardiometabolic risk. The goal of this pilot study was to determine if SUA is increased in patients with metabolic syndrome and to investigate correlations between SUA and individual cardiometabolic risk factors.

**Findings:**

Volunteers between the ages of 18 and 65 without conditions known to affect serum uric acid levels were recruited. Height, weight, blood pressure and waist circumference were measured and a full lipid panel along with fasting blood glucose was obtained. Saliva samples were collected and uric acid levels were determined. 78 volunteers, 35% of whom had metabolic syndrome, completed the study. SUA was significantly elevated in patients with metabolic syndrome (p=.002). The incidence of metabolic syndrome in the 4^th^ quartile for SUA was 67% compared to 25% in quartiles1-3 combined. Significant correlations were seen between SUA and systolic blood pressure (r=.440, p=.000), diastolic blood pressure ( r=.304, p=.007), waist circumference (r=.332, p=.003), BMI ( r=.269, p=.018), fasting blood glucose ( r=.341, p=.002), triglycerides (r=.410, p=.000), HDL ( r=.237, p=.036) and the number of cardiometabolic risk factors present (r=0.257, p=.023).

**Conclusions:**

These results suggest that SUA may be a useful biomarker for noninvasive monitoring of cardiometabolic risk. Larger studies are needed to validate this approach.

## Findings

### Introduction

The impact of obesity on health outcomes can be seen in the rising incidence of diabetes and cardiovascular disease, particularly in rural states such as West Virginia (WV). Two-thirds of adults in WV are obese or overweight and over 12% already have diabetes [1]. In addition, 7.5% of WV adults have had a heart attack compared to a national average of 4.4% [1]. Because these diseases can be prevented or delayed by lifestyle interventions, novel approaches to identifying patients at the greatest risk are needed. One tool for identifying high risk patients is screening for metabolic syndrome, a combination of risk factors including increased waist circumference, high blood pressure, elevated triglycerides (TRG), reduced high density lipoproteins (HDL), and impaired fasting blood glucose (IFG) [2]. Patients with at least three of these factors are classified as having metabolic syndrome and it is critical that such patients be identified so these modifiable risk factors can be improved.

While the importance of monitoring and reducing cardiometabolic risk is clear, there are significant challenges to addressing this issue, especially in rural areas. These challenges include lack of access, cost, and resistance to invasive monitoring procedures [3]. Noninvasive methods for assessing disease risk could increase participation in screening and treatment programs and improve adherence to dietary and lifestyle interventions. One approach to noninvasive monitoring is the use of salivary biomarkers. Several *serum* biomarkers associated with cardiometabolic risk including C-reactive protein, adiponectin, and uric acid can also be detected in saliva [4-7]. Of particular interest is the fact that a linear relationship between salivary and serum uric acid levels has been observed suggesting that saliva may be a useful surrogate for blood testing [7,8], Soukup et al unpublished results]. While numerous studies have demonstrated an association between serum uric acid, metabolic syndrome and cardiovascular disease risk, its role is not well understood [reviewed in [9,10]. Uric acid is the terminal degradation product of purine catabolism and contributes to the antioxidant capacity of both blood and saliva. However, the enzyme responsible for its production also generates free radicals and several studies have shown that uric acid can act as a pro-inflammatory and pro-oxidant agent [reviewed in [10]. Regardless of whether it plays a causative role or is an indicator of metabolic disturbances, uric acid may be a useful biomarker for identifying high risk patients and monitoring the response to lifestyle interventions. The purpose of this pilot study was to investigate the feasibility of a noninvasive approach by determining if salivary uric acid is elevated in adults with metabolic syndrome.

### Study design

This study (NCT01086137) was approved by the WVSOM Institutional Review Board. Volunteers between the ages of 18 and 65 were recruited from Greenbrier and surrounding counties in WV. Volunteers were not compensated but were given the option to receive blood test results free of charge. Subjects with conditions known to affect salivation, uric acid, or inflammatory biomarkers including gout, renal insufficiency, Sjogrens syndrome, autoimmune disease, active infection, type II diabetes and pregnancy were excluded. A medical history was taken, and information regarding oral health status, family history, medications, supplements and smoking status was collected. Subjects then provided a saliva sample using the passive drool method and flow rates were determined [11]. Height, weight, blood pressure and waist circumference were measured using standard procedures and a full lipid panel along with fasting blood glucose was obtained with a fingerprick sample using the Cholestech LDX instrument [12]. The presence of metabolic syndrome was determined using the new worldwide consensus criteria [13]. Waist circumference cutpoints in these criteria are country specific with US (AHA/NHLBI) guidelines suggesting that the lower values of 94cm in men and 80cm in women are appropriate for populations with an increased likelihood of insulin resistance [13]. Since the majority of subjects in this study were obese or overweight and had a family history of T2DM and nearly 20% had impaired fasting glucose, these lower cutpoints were used. Analysis using the higher ATPIII cutpoints resulted in re-classification of two subjects and very similar results (data not shown).

### Uric acid measurement

Saliva samples were thawed on ice and the mucins were removed through centrifugation [11]. Supernatants were then applied to a spin column in order to remove peroxidase enzymes that could interfere with the uric acid measurement. Samples contaminated with blood were excluded from analysis. Salivary uric acid (SUA) concentration was determined using the enzymatic uric acid assay reagent from Pointe Scientific (Canton, MI) in a 96-well format. This method had intra- and inter-assay variability of less than 5% and average recovery of 104%.

### Statistical analysis

All statistical analyses were performed using PASW version 17 software. Normality was assessed using skewness and kurtosis and values for BMI, salivary flow rate, triglycerides, and serum HDL were normalized with a log transformation. Mean values were compared using unpaired t-tests. Categorical values were compared using the chi-squared test. Correlations were investigated by calculating Pearson productmoment correlation coefficients. Spearmans rho was used to investigate correlations between salivary uric acid and the number of cardiometabolic risk factors present. Partial correlations adjusting for sex were also calculated. Statistical significance was set at p<0.05.

## Results

**Figure 1:**
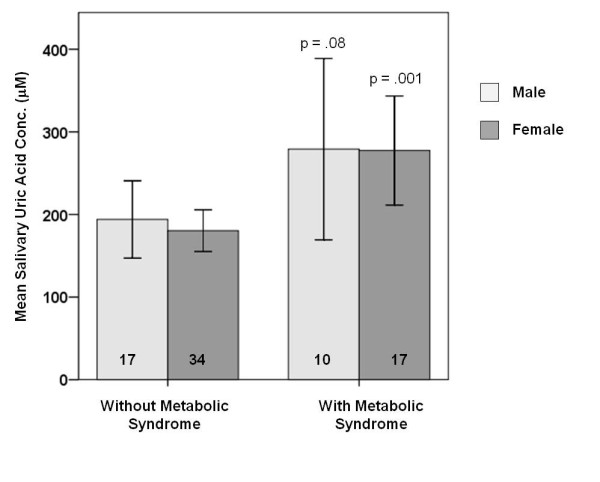
**Salivary uric acid levels in male and female subjects with or without metabolic syndrome. **Error bars represent 95% confidence intervals. The number of subjects is shown at the bottom of each bar.

**Figure 2:**
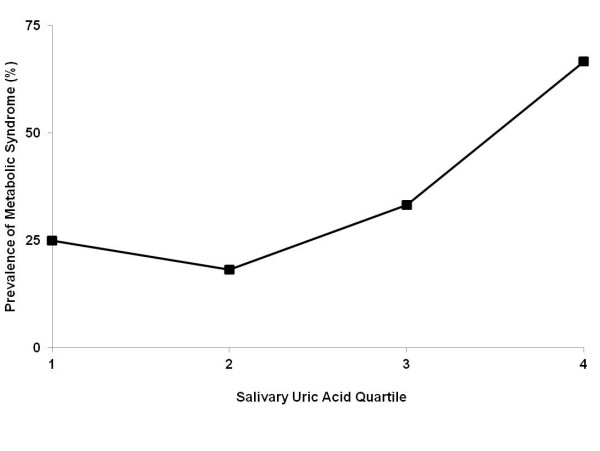
**The prevalence of metabolic syndrome by uric acid quartile. **The number of subjects in quartiles one through four were 20, 22, 18 and 18 respectively.

Samples from 78 volunteers who completed the study were included in the analysis. Of these, 35% had metabolic syndrome. Subject characteristics and biomarker levels are summarized in Table 1. SUA levels were significantly elevated in patients with metabolic syndrome (p=.002) independent of salivary flow rate. No significant differences were seen between overall SUA levels in men vs. women, or people with a history of periodontal disease vs. those without. However, the relationship between SUA and metabolic syndrome was stronger in women ( p=.001) than in men ( p=.08) (Figure 1). The overall incidence of metabolic syndrome in the 4^th^ quartile for SUA was 67% compared to 18-33% in quartiles 13 (Figure 2). Significant positive correlations were seen between SUA and systolic blood pressure (r=.440, p=.000), diastolic blood pressure ( r=.304, p=.007), waist circumference (r=.332, p=.003), BMI ( r=.269, p=.018), fasting blood glucose ( r=.341, p=.002), triglycerides (r=.410, p=.000) and the number of cardiometabolic risk factors present (r=0.257, p=0.023). SUA levels were negatively correlated with HDL ( r=.237, p=.036). These relationships remained significant when partial correlations were calculated after adjusting for sex..

**Table 1:** Characteristics of the study population and biomarker levels

	**Total** **(n=78)**	**Without Metabolic Syndrome (n=51)**	**With Metabolic Syndrome(n=27)**	**p**
**Age**	43.5±1.8	38.0±15.5	53.7±10.2	.000
**Gender (male/female)**	27/51	17/34	10/17	.745
**History of periodontal disease (%)**	21.8%	13.7%	37.0%	.018
BMI*	26.3±1.2	24.5±1.2	31.6±1.1	.000
**Waist Circumference (cm)**	91.5±14.3	84.9±10.6	103.9±11.9	.000
Triglycerides (mg/dL)*	97.7±1.8	79.4±1.5	147.9±2.0	.000
HDL(mg/dL)*	45.7±1.4	50.1±1.3	37.2±1.3	.000
**Total Cholesterol (mg/dL)**	189.1±41.1	176.9±34.3	212.1±43.5	.000
**Glucose (mg/dL)**	91.3±10.1	88.6±8.0	96.4±11.8	.001
**Systolic BP (mmHg)**	120.2±13.6	114.8±11.1	130.4±12.3	.000
**Diastolic BP (mmHg)**	78.5±10.3	74.4±7.2	86.1±11.1	.000
**Salivary Uric Acid (M)**	217.2±110.3	184.9±78.4	278.1±135.3	.002
Salivary flow rate (mL/min)*	0.46±0.72	0.48±0.47	0.43±0.53	.468

Values are means+SD.

* P values obtained after log_10_ transformation. Geometric means are shown

## Discussion

In recent years, significant advances have been made toward the validation of salivary biomarkers for disease detection. Methods using oral fluids for diagnosis of disorders including myocardial infarction, periodontal disease, and cancer are being investigated [14]. Because both metabolic syndrome and T2DM have been linked to periodontal disease, oral health care providers have an important role in identifying high risk patients, making oral diagnostics a good fit for these indications. Several studies have shown that serum uric acid levels are elevated in patients with metabolic syndrome [reviewed in 9, 10]. This study demonstrates that salivary uric acid is also elevated in patients with metabolic syndrome and correlates with several cardiometabolic risk factors including blood pressure, triglyceride levels, HDL and fasting blood glucose. As seen in other studies looking at serum, the relationship between SUA and metabolic syndrome was stronger in females than in males [15-17]. While larger studies are needed to delineate the factors contributing to SUA levels and their association with metabolic syndrome, our results suggest that that SUA may be a viable noninvasive biomarker for monitoring cardiometabolic risk particularly in women.

Limitations of this pilot study include the small sample size, the fact that oral health status was self-reported, and the absence of detailed information regarding emotional status, nutrition and alcohol consumption which could affect salivation or uric acid levels. However, the results suggest that this approach should be further explored. Follow-up studies to validate SUA as a biomarker of metabolic syndrome should determine not only its predictive value but also its ability to improve models consisting of other noninvasive measures such as waist circumference. The inclusion of additional salivary biomarkers might further improve these models and studies investigating other salivary changes associated with obesity and metabolic syndrome have recently been published [18,19]. These candidate markers could be included in future studies. The ability to monitor cardiometabolic risk using simple, noninvasive testing should help overcome barriers to screening and may also improve adherence to dietary and behavioral treatment programs.

## Competing interests

The authors declare that they have no competing interests.

## Authors contributions

MS assisted with data collection and analysis, completed the biomarker assays, and drafted the manuscript. IB, BI and DR assisted with data collection and uric acid analysis. LR participated in the study design and completed the statistical analysis. AH, EB and AN assisted with study design and coordination, drafting of the clinical protocol, and data collection and analysis. KB conceived of the study, assisted in its design, coordination, and analysis, and helped to draft the manuscript. All authors read and approved the final manuscript.
